# Resource heterogeneity and the evolution of public goods cooperation

**DOI:** 10.1002/evl3.158

**Published:** 2020-02-04

**Authors:** Peter Stilwell, Siobhan O'Brien, Elze Hesse, Chris Lowe, Andy Gardner, Angus Buckling

**Affiliations:** ^1^ Department of Biosciences University of Exeter Penryn TR10 9FE United Kingdom; ^2^ Institute of Integrative Biology University of Liverpool Liverpool L69 7ZB United Kingdom; ^3^ School of Biology University of St Andrews St Andrews KY16 9TH United Kingdom

**Keywords:** Cooperation, evolution, microorganisms, models, resource heterogeneity, siderophores

## Abstract

Heterogeneity in resources is a ubiquitous feature of natural landscapes affecting many aspects of biology. However, the effect of environmental heterogeneity on the evolution of cooperation has been less well studied. Here, using a mixture of theory and experiments measuring siderophore production by the bacterium *Pseudomonas aeruginosa* as a model for public goods based cooperation, we explore the effect of heterogeneity in resource availability. We show that cooperation in metapopulations that were spatially heterogeneous in terms of resources can be maintained at a higher level than in homogeneous metapopulations of the same average resource value. The results can be explained by a positive covariance between fitness of cooperators, population size, and local resource availability, which allowed cooperators to have a disproportionate advantage within the heterogeneous metapopulations. These results suggest that natural environmental variation may help to maintain cooperation.

**Impact Summary**

Heterogeneity in resource availability is a ubiquitous feature of natural landscapes that has been shown to influence population dynamics and community structure. The effect of resource heterogeneity on the evolution of cooperation has been less well explored. Resource availability has been shown to affect the cost of cooperation but will also affect population size. The covariance between population size and the cost of cooperation is the subject of our investigations in this paper. We first develop an analytical model formalizing our hypothesis and then conduct experiments to investigate the role of resource heterogeneity on pyoverdine production in the bacterium *Pseudomonas aeruginosa*.

Our analytical model shows that under hard selection, a greater degree of cooperation is favored in heterogeneous environments, as the populations in which cooperation is favored contribute more ancestry to future generations. Under soft selection, the contribution of populations to future generations is unlinked from their current productivities, and so resource heterogeneity has no impact on the level of cooperation.

In a series of in vitro experiments, we first confirm that resource levels have an effect on *P. aeruginosa* cooperator fitness in the short term, before conducting a longer term evolution experiment that supports the prediction that heterogeneous environments support higher levels of cooperation than homogeneous environments.

These results emphasize the role of abiotic variation in the evolution and maintenance of cooperation.

The amount of resource available in an environment plays a key role in the evolution of cooperation. Higher resource availability can favor increased cooperation by reducing the relative costs associated with the behavior (Bednekoff and Woolfenden 2003; Brockhurst et al. 2008; Connelly et al. 2017; Dumas and Kümmerli 2012; Xavier et al. 2011), likely due to a trade‐off between growth and investment into cooperation (Foster 2004), that is, cooperation requires a smaller proportion of resources that could otherwise be invested in growth. Despite the clear importance of resource availability in the evolution of cooperation, we currently lack an understanding of the importance of a ubiquitous feature of natural environments: heterogeneity. It is well established that environmental and genetic heterogeneity in a geographic landscape will alter the (co)evolutionary trajectory of the populations therein (Hesse et al. 2015; Thompson, 1999, 2005; Vogwill et al. 2009). Here, we investigate the impact of resource heterogeneity on the evolution of cooperative interactions in the opportunistic bacterial pathogen *Pseudomonas aeruginosa*.

Recent theory using numerical simulations suggests that heterogeneity in resource availability increases cooperation, although the mechanisms underlying these results are unclear (Kun and Dieckmann 2013). One general effect of resource heterogeneity on cooperation likely stems from the positive covariance between cooperation, local resource availability, and density: per capita resource‐rich patches support greater population growth rates and individuals are more likely to be cooperators because the relative cost of cooperation is low (Brockhurst et al. 2008). Where the magnitude of dispersal acting to redistribute genotypes across the geographic mosaic is in proportion to the population size of a patch, that is, the population is under hard selection (Fig. 1), cooperation might then be maintained to a higher degree in heterogeneous environments.

**Figure 1:**
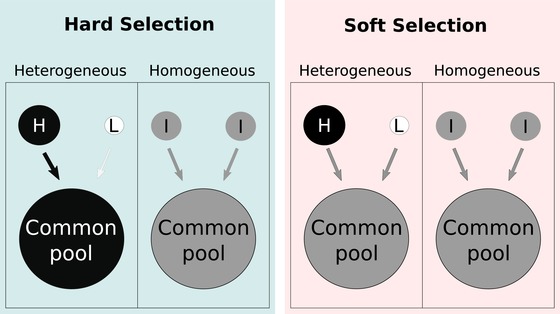
Resource heterogeneity can maintain cooperation under hard selection. H, L, and I correspond to high, low, and intermediate resource levels, respectively. Level of cooperation is depicted by variation along a grayscale, where black circles contain more cooperators and white contain more cheats. Productivity (i.e., contribution to the common pool) is represented by arrow thickness and tone. Under hard selection, resource rich (and hence more productive populations) are overrepresented in the pool. Under soft selection, contribution to the pool is not affected by productivity.

Cooperation based around the production of public goods is ubiquitous in microorganisms, usually in the form of a product secreted into the environment, the benefit from which may be received by individuals in the vicinity. Siderophores are one such example. Iron is necessary for the growth and survival of almost all life on earth (Andrews et al. 2003), and in iron‐limited conditions many microorganisms will secrete siderophores into the environment. These molecules have a high affinity for iron, and once bound can be taken‐up by the microorganism usually via receptor binding (Hider and Kong 2010). Pyoverdine, the primary siderophore produced by certain *Pseudomonas* species, has been extensively studied in the context of social evolution (Brockhurst et al. 2006; Buckling et al. 2007; Butaitė et al. 2017; Harrison 2013; Harrison and Buckling 2007; Harrison et al. 2006; Julou et al. 2013; Köhler et al. 2009; Kümmerli et al. 2010; Ross‐gillespie et al. 2007; Zhang and Rainey 2013). As the individual bacterium producing the pyoverdine is not necessarily the beneficiary, production can be considered a cooperative yet exploitable public good. Nonpyoverdine producing mutants readily evolve in the lab, and in iron‐limited conditions these increase in frequency beyond their wild‐type counterparts and can be considered social cheats (West and Buckling 2003). Previous work has shown that selection for siderophore cheating is reduced when resource levels (nutrient media [Brockhurst et al. 2008], iron, and exogenous siderophores [Dumas and Kümmerli 2012]) are increased. The latter study also considered spatial resource heterogeneity, but the experimental design meant that it was not possible to disentangle an effect of resource heterogeneity from that of mean levels of resources.

Here, we investigate how resource heterogeneity may affect cooperation through the simple mechanism of a positive covariance of population size and cooperation. We first develop an analytical model to formalize the hypothesis, and then conduct experiments to investigate the role of resource heterogeneity on pyoverdine production by the bacterium *P. aeruginosa*, using a combination of competition experiments between isogenic cooperating and noncooperating strains and experimental evolution.

## Theory

### SINGLE POPULATION

Assume that bacterial growth comprises a basic rate  γ, proportional to both resource and siderophore availability, and an accelerating growth cost of investment into siderophore production. Specifically, γ = *Ry* – *x* ^2^, where *R* is the availability of general resources (e.g., carbon) used for both growth and public goods production (the same value being experienced by all cells in the population), *y* is the availability of siderophore (being the average siderophore production of the focal cell's neighbors, which varies from cell to cell) and *x* is the cell's own investment into siderophore production (which also varies from cell to cell). These assumptions mean that the relative cost (i.e., proportional reduction in growth) of producing siderophores decreases with increasing resource availability (Brockhurst et al. 2008; Sexton and Schuster 2017), and this particular functional form has been chosen so as to ensure that both the selection differential and the evolutionary equilibrium level of cooperation are linear functions of resource availability. We consider the effect of nonlinearity in a later section.

A focal cell's fitness may then be written as *w_R_* = exp(*Ry* – *x* ^2^) and the average fitness of all cells in the population may be written as ${\bar{w}}_{R}=\exp\left(Rz-z^{2}\right)$, (where *z* is the average siderophore production), on the simplifying assumption of vanishingly little variation in siderophore production. Accordingly, relative fitness may be defined as $W_{R}=w_{R}/\overset{\prime }{w_{R}}$ and the criterion for natural selection to favor an increase in siderophore production in this population is $\frac{dW_{R}}{dx}{|}_{x=y=z}>0$. From the chain rule, $\frac{dW_{R}}{dx}{|}_{x=y=z}\;=\frac{\partial W_{R}}{\partial x}{|}_{x=y=z}\;+\frac{\partial W_{R}}{dy}{|}_{x=y=z}\;\frac{dy}{dx}{|}_{x=y=z}=Rr-2z$, where $r=\frac{dy}{dx}{|}_{x=y=z}$ is the average relatedness between a given cell and those cells that make use of its siderophores (Taylor and Frank 1996). The stable level of siderophore production z* therefore satisfies *Rr* – 2z* = 0, that is, z* = *Rr*/2.

### METAPOPULATION

Now consider a metapopulation in which each constituent population may have a different level of resource availability. Assuming that resource availability varies continuously, the probability density of populations having resource availability *R* may be denoted *p_R_*, satisfying *p_R_* ≥ 0 and $\int_{R\min}^{R\max}p_{R}dR=1$. Natural selection favors an increase in siderophore production across the whole metapopulation if $\int_{R\min}^{R\max}c_{R}{{}_{x}}_{=y=z})dR>0$, where *c_R_* is the proportion of the ancestry of future generations that is contributed by populations with resource availability *R* (Taylor and Frank 1996).

### HARD AND SOFT SELECTION

The stable levels of siderophore production favored by selection in this metapopulation context will likely depend on whether selection is “hard” or “soft” (Fig. 1) (Christiansen 1975; Wallace, 1968, 1975). In the context of soft selection, all populations contribute the same ancestry to future generations, such that *c_R_* = *p_R_*. Accordingly, $\int_{R\;\min}^{R\;\max}c_{R}dW_{R}/\mathrm{dx}{|}_{x=y=z}dR=\int_{R\;\min}^{R\;\max}p_{R}\left(Rr-2z\right)dR=\bar{R}r-2z$, and hence $z^{*}=\bar{R}r/2$, where $\bar{R}$ is the average availability of resources across the metapopulation. In other words, heterogeneity in resource availability has no impact on the level of competition.

In the context of hard selection, each population contributes ancestry to future generations in proportion to its overall growth, such that $c_{R}=p_{R}\;{\bar{w}}_{R}/\bar{w},$ where $\bar{w}=\int_{R\;\min}^{R\;\max}p_{R}\;w_{R}dR$ is the average of fitness across the metapopulation. Accordingly,

$$\begin{array}{ccc} & & \int_{R\;\min}^{R\;\max}c_{R}dW_{R}/\mathrm{dx}{|}_{x=y=z}dR=\int_{R\;\min}^{R\;\max}\left(p_{R}/\bar{w}\right)\;\exp\;\left(\left(R-z\right)z\right)\\ & & \left(Rr-2z\right)dR\approx \exp\left(\left(\bar{R}-z\right)z\right)\;(\bar{R}r-2z-1/2(z(2z^{2}-r\\ & & \left(2+\bar{R}z\right)))\sigma_{R}^{2})/\bar{w} \end{array}$$

and hence $z^{*}\approx \left(Rr/2\right)\left(1+\left(r\sigma_{R}^{2}/2\right)\right)$. That is, variation in resource availability across the metapopulation $\left(\sigma_{R}^{2}>0\right)$ favors a greater degree of cooperation (higher z*) owing to those populations in which cooperation is most favored contributing more ancestry to future generations.

### NONLINEAR IMPACT OF RESOURCES ON COOPERATION

The model formulation above assumes a positive linear relationship between resource levels and selection for cooperation. This relationship can of course be nonlinear. Nonlinearity can both increase the effect of heterogeneity on selection for cooperation if cooperation is an accelerating function of resources and can result in heterogeneity selecting against cooperation when cooperation is a decelerating function of resources. However, these findings are an inevitable and trivial consequence of nonlinearity, where the mean of two points will differ from the intermediate point (a corollary of Jensen's Inequality; Jensen 1906).

For example, if we consider that growth is given by γ = *R^a^y* – *x* ^2^, where the exponent parameter *a* allows for nonlinearity (*a* < 1 corresponds to diminishing returns of resources on growth rate, *a* = 1 corresponds to the linear returns scenario considered above, and *a* > 1 corresponds to accelerating returns of resources on growth rate), then in (1) the absence of resource heterogeneity natural selection favors a level of cooperation z* = *rR^a^*/2; (2) in the presence of resource heterogeneity and soft selection, natural selection favors a level of cooperation $z^{*}\approx \left(r\;{\bar{R}}^{a}/2\right)\left(1+1/2\;a\left(a-1\right){\bar{R}}^{-2}\sigma_{R}^{2}\right)$, which is less than that obtained in the absence of resource heterogeneity when *a* < 1 and is greater than that obtained in absence of resource heterogeneity when *a* > 1; and (3) in the presence of resource heterogeneity and hard selection, natural selection favors a level of cooperation $z^{*}\approx \left(r\;{\bar{R}}^{a}/2\right)\;\left(1+\left(a\left(a-1+ar\;{\bar{R}}^{2a}\right)/\left(2\;{\bar{R}}^{2}\right)\right)\sigma_{R}^{2}\right)$, which is greater than obtained under soft selection, on account of it incorporating not only the nonlinearity effect but also the between‐patch productivity differences effect.

## Materials and Methods

### BACTERIAL STRAINS

*Pseudomonas aeruginosa* strain PAO1, a wild‐type, siderophore producing strain, was used as a cooperator (referred to as PAO1 WT cooperator). A siderophore‐negative strain, PAO1∆pvd∆pchEF (Ghysels et al. 2004), which knocks out production of both pyoverdine (the primary siderophore of PAO1) and pyochelin (a weaker iron chelating molecule produced by PAO1) (Cornelis 2010), marked with *LacZ* (using the plasmids and protocol described in Choi et al. 2006) was used as a nonproducing cheat (referred to as PAO1 *LacZ* cheat). *LacZ* gave PAO1∆pvd∆pchEF a blue pigment, so that it could be easily distinguished from PAO1 on Lysogeny Broth agar supplemented with 90 μg/mL 5‐bromo‐4‐chloro‐3‐indolyl‐β‐d‐galactopyranoside (X‐gal). This marker confers no fitness cost to the cheat (O'Brien et al. 2017). Replicates were inoculated with overnight cultures grown each from single colonies, picked from streaks of glycerol freezer stocks.

### SINGLE POPULATION COMPETITION AND DENSITY EXPERIMENTS AS A FUNCTION OF RESOURCE AVAILABILITY

Three media containing different levels of resource were prepared: “high,” a 1:4 dilution of casamino acids medium (CAA: 5 g casamino acids, 1.18 g K_2_HPO_4_•3H_2_O, 0.25 g MgSO_4_•7H_2_O in 1 L H_2_O) in M9 salts (M9 salts: 12.8 g Na_2_HPO_4_.7H_2_O, 3 g KH_2_PO_4_, 0.5 g NaCl, 1 g NH_4_Cl, in 1 L Millipore H_2_O); “low,” a 1:16 dilution of CAA in M9 salts; and “intermediate,” a 1:1 mix of “high” and “low” media. To render environments iron limited, all media were supplemented with 100 µg/mL human apo‐transferrin (an iron chelator) and sodium bicarbonate at a final concentration of 20 mM (necessary for iron chelator activity, Meyer et al. 1996). Overnight cultures of PAO1 WT cooperator and PAO1 *lacZ* cheat grown at 37 °C, shaking at 180 r.p.m. in 6 mL “intermediate” culture medium, were diluted to OD600 ∼0.1 in M9 salts, and 30 µL of each was co‐inoculated into 12 replicates of 6 mL of each of the resource media. Populations were plated both at the beginning of the experiment, and at 48 hours postinoculation (h.p.i.) on KB agar (Kings B medium [10 g glycerol, 20 g proteose peptone No.4, 1.5 g MgSO_4_, 1.5 g K_2_HPO_4,_ in 1 L Millipore H_2_O] supplemented with 12 g bacteriological agar and X‐Gal at a final concentration of 50 µg/mL). Colonies were enumerated according to their *LacZ* phenotype, and the relative fitness *(W)* of the cooperative strategy was estimated by taking the ratio of strain's Malthusian parameters (*m*) (the natural log of a strain's final density over its starting density). To test the effect of resource availability on cooperation, relative fitness estimates were compared using a one‐way analysis of variance (ANOVA) and post hoc Tukey–HSD contrasts, with α = 0.05. To test whether the relationship between fitness and resource availability was roughly linear, relative fitness in the intermediate group was compared to the mean of the high and low groups using one‐sample *t*‐tests. One‐sample *t*‐tests were also used to compare relative fitness in each group to a hypothesized mean of 1 (where cooperator and cheat fitness is equal) to quantify whether cooperation had declined in all groups.

We also determined how the density of wild‐type bacteria alone was affected by the three resource levels, with log10‐transformed density compared using a one‐way ANOVA and post hoc Tukey–HSD contrasts.

### COMPETITION EXPERIMENTS IN HETEROGENEOUS AND HOMOGENOUS METAPOPULATIONS

Two treatment groups, corresponding to the heterogeneous and homogeneous environments, consisted each of 12 replicate pairs of microcosms, the heterogeneous treatments containing 12 high‐ and 12 low‐resource availability environments, and the homogeneous treatment containing 12 pairs of intermediate resource availability environments. “High,” “low,” and “intermediate” resource availability media were created as described previously. Note that 6 mL of the relevant media was aliquoted into 30 mL glass microcosms. Each microcosm was coinoculated with 30 µL of PAO1 WT cooperator and 30 µL of PAO1 *lacZ* cheat from overnight cultures grown in “homogeneous” treatment media. All replicates were plated after inoculation on KB agar with X‐Gal to verify initial densities. At 48 h.p.i., the matched‐pair high‐ and low‐resource cultures in the heterogeneous treatment were mixed, as were the paired cultures in the homogeneous treatment. Sixty microliters (i.e., 1% by volume) of these mixes were transferred into two fresh microcosms according to treatment. At 96 h.p.i, all paired cultures were again mixed before being plated on KB agar with X‐Gal. Cooperator fitness estimates (*W*) were calculated as in the single population competition experiment and were then compared against a hypothesized mean of 1 using one‐sample *t*‐tests.

### EXPERIMENTAL EVOLUTION IN HETEROGENEOUS AND HOMOGENOUS METAPOPULATIONS

We performed experimental evolution to assess how resource heterogeneity shaped the evolution of de novo siderophore cheats. The setup of this experiment was identical to that of the meta‐population competition experiment described above, except each microcosm was inoculated with 60 µL of only PAO1 WT cooperator from overnight culture in homogeneous treatment media. Every 48 hours, paired replicate microcosms were mixed and transferred into new media according to treatment. This was repeated for 20 transfers (∼150 generations). At the end of the experiment, cultures were frozen in 20% glycerol and stored at –80 °C for later assay of siderophore iron chelating activity.

### PER CAPITA IRON CHELATOR ACTIVITY ASSAY

Note that 60 µL of thawed glycerol stock of the 20th transfer was inoculated into iron‐limited CAA medium and grown overnight at 37 °C shaking at 180 r.p.m. From this 1 mL of culture was centrifuged at 14,000 rpm in a benchtop microcentrifuge. The iron chelator activity in the supernatant was determined using the chrome azurol S (CAS) assay described in Schwyn and Neilands (1987): 100 µL of supernatant from centrifuged culture was mixed with 100 µL of CAS solution in a 96‐well plate, and incubated for 1 hour in the dark at room temperature. Per capita iron chelator activity is given by: 1 – (*A_i_*/*A*_ref_)/density *i*, where *A_i_* is the absorbance of the *i*th sample at 630 nm, and *A*_ref_ is the absorbance at 630 nm of a reference CAS reaction carried out on the media in which the culture was grown. Density is the sample absorbance at 600 nm. To ensure the absorbance at 600 nm was measured in the linear range, a 1 in 10 dilution of the sample was used to estimate culture density. To determine between‐clone variation in siderophore production, we repeated these assays on 24 randomly picked colonies per population, as described above for whole populations.

For the whole‐population CAS assays, we used ANOVA to determine whether *per capita* iron chelator activity (a measure of siderophore production and hence cooperation) differed between ancestral WT PAO1 (i.e., at transfer 0), the heterogeneous and homogeneous treatments at 20th transfer. Post hoc Tukey–HSD was used to determine the significance of differences between treatment groups. For the clonal level CAS assays, we tested for the effect of resource heterogeneity on mean and between‐clone variation in siderophore production by fitting a linear mixed effects (LME) model (“*lmer*” function in “*lme4*” package, Bates et al. 2015) on log‐transformed siderophore data with resource treatment as fixed explanatory variable and random intercepts fitted for individual populations. All analyses were conducted using R version 3.6.1.

## Results

### SINGLE POPULATION COMPETITION EXPERIMENTS

**Figure 2:**
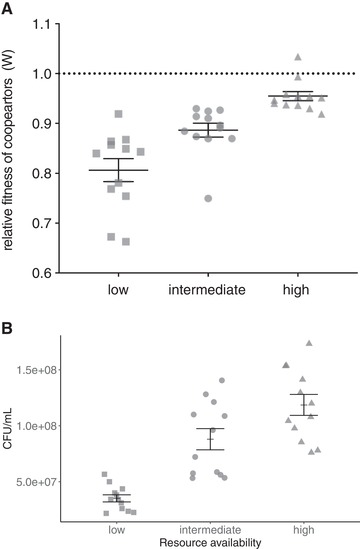
(A) Fitness of cooperators relative to cheats in the three medias over 48 hours of growth. Bars show mean ± SEM. (B) Densities after 48 hours growth in the three media. Bars show mean ± SEM.

We first established how short‐term levels of cooperation varied as a function of resource availability. The amount of resource had a significant effect on *P. aeruginosa* siderophore cooperator fitness (*F*_2,33_ = 20.51, *P* < 0.0001) when in competition with cheats: cooperators had higher relative fitness in high‐resource availability media than in both intermediate (Tukey's HSD, *P* = 0.0156), and low‐resource availability media (Tukey's HSD, *P* < 0.001), and cooperators in intermediate resource availability media had higher fitness than in low‐resource availability media (Tukey's HSD, *P* = 0.004; Fig. 2A). Cooperator fitness in intermediate resource availability media was not different to that of the mean of cooperator fitness in high‐ and low‐resource availability media (one‐sample *t*‐test against a hypothesized mean of 0.88, *t*_11_ = 0.42, *P* = 0.68). This suggests levels of cooperation are proportional to resource availability, which is a key assumption of our model.

Despite the increasing benefits of cooperation with increase resource level, cooperator fitness in each treatment remained significantly lower than that of cheats (one‐sample *t*‐tests against a hypothesized mean of 1; high‐resource availability, *t*_11_ = −5.03, *P* = 0.001; intermediate‐resource availability, *t*_11_ = −8.12, *P* < 0.001; low‐resource availability, *t*_11_ = −8.41, *P* < 0.001; Fig. 2A).

Finally, we confirmed that cooperator population densities increased with increasing resource levels after 48‐hour growth (*F*_2,33_ = 28.65, *P* < 0.0001, Tukey's HSD, “high” compared with “intermediate,” *P* = 0.025; “high” compared with “low,” *P* < 0.0001; “intermediate” compared with “low,” *P* = 0.0001; Fig. 2B). This satisfies the second key assumption of our model.

### COMPETITION EXPERIMENTS IN HETEROGENEOUS AND HOMOGENOUS METAPOPULATIONS

**Figure 3:**
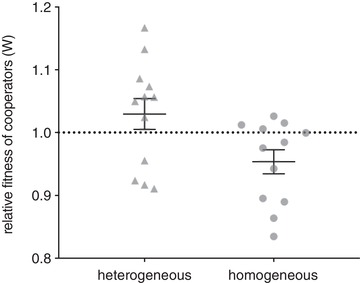
Fitness of cooperators relative to cheats in spatial homogeneity and heterogeneity after two transfers. Bars show mean ± SEM.

We next conducted competition experiments between isogenic cooperators and cheats as above but under metapopulation conditions. We found that wild‐type siderophore‐producing cooperators had higher fitness when in heterogeneous environments than in homogeneous environments (*t*‐test, *t*_22_ = −2.426, *P* = 0.0239; Fig. 3), though neither the homogeneous nor heterogeneous cooperators had fitnesses different to 1 (homogeneous, *t*‐test, *t*_11_ = −2.408, *P* = 0.0695; heterogeneous, *t*‐test, *t*_11_ = 1.200, *P* = 0.0511).

### EXPERIMENTAL EVOLUTION IN HETEROGENEOUS AND HOMOGENOUS METAPOPULATIONS

**Figure 4:**
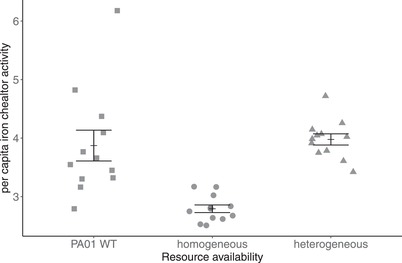
Per capita iron chelator activity (a measure of siderophore production and cooperation) in PA01 WT and after 20th transfer in the homogeneous and heterogeneous treatment groups. Bars show mean ± SEM.

Finally, we conducted an evolution experiment to determine whether the effect of resource heterogeneity on the costs of cooperation and population density would maintain cooperation. We found that resource heterogeneity had a significant effect on the level of public goods cooperation (Fig. 4; *F*_33,1_ = 15.59, *P* < 0.0001). Post hoc tests revealed that the level of cooperation in the heterogeneous treatment at 20th transfer was not different to that of PAO1 WT (Tukey’ HSD, *P* = 0.896), although the level of cooperation in the homogeneous treatment had dropped compared with both that of the WT (Tukey's HSD, *P* = 0.0002) and the heterogeneous treatment (Tukey's HSD, *P* < 0.0001).

**Figure 5:**
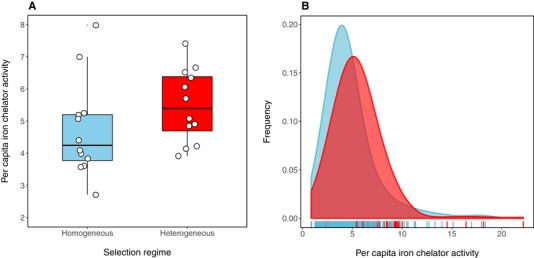
The effect of resource heterogeneity (blue = homogeneous and red = heterogeneous) on (A) mean (white circles is individual populations, *n =* 12 per treatment) and (B) distribution of siderophore production in evolved *P. aeruginosa* populations after 20 transfers.

Analyses of clone‐level siderophore production revealed the distribution of siderophore production to be largely unimodal, as opposed to bimodal with distinct groups of high and low producers (Fig. 5; note that the same qualitative patterns were observed for individual replicates). Mean siderophore production was marginally greater in heterogeneous environments (LME: ${}_{1}^{2}$= 3.53, *P* = 0.06) and varied substantially across populations between treatments (explaining 27% of the total variance). The marginally nonsignificant differences in mean siderophore production between treatments in these assays (in contrast to the whole‐population assays, where differences were highly significant) can be explained by the much smaller sample sizes (24 clones per population) in the former. These findings provide further support for our model, which predicts single optimal strategies of siderophore production, as opposed to, for example, divergence into co‐operators and cheats. This prediction could necessarily not be tested by the competition experiments between cooperators and cheats.

## Discussion

We investigated the effect of spatial heterogeneity in resource availability—while maintaining the same mean level of resources—on the evolution of cooperation. We first developed an analytical model showing that under hard selection, where fitness is determined relative to all members within the metapopulation, resource heterogeneity can promote higher levels of cooperation. We found support for this prediction in both a short‐term competition experiment and a long‐term evolution experiment using a model cooperative trait: siderophore production by *P. aeruginosa*.

The mechanism underpinning our theoretical and experimental results is the positive covariance between cooperation and productivity resulting from variation in resource availability. Specifically, in a meta‐population consisting of patches that vary with respect to their resource availability, high‐resource availability patches, containing a higher proportion of cooperators, contribute a greater number of individuals to the next generation. This results in a higher frequency of cooperators across metapopulations. There are reasons to believe this may be of general importance for the evolutionary maintenance of cooperation: heterogeneity in resource availability is ubiquitous (Rosenzweig 1995), and many species exist as metapopulations, providing opportunities for hard selection (Hanski 1999). However, the mechanism also relies on positive linear relationships between selection for cooperation and resource availability. Simple extensions of our model show that nonlinear relationships could swamp the covariance mechanism we address here, potentially resulting in cooperation being hindered or helped under both soft and hard selection for a somewhat trivial reason. Specifically, nonlinearities require that mean cooperator fitness will differ between homogeneous and heterogeneous resource environments, with heterogeneous environments having greater fitness if cooperator fitness is an accelerating function of resource levels, and vice versa when there is a decelerating function. Such nonlinearities could arise when, for example, smaller groups are more cooperative when interactions are more identifiable (Boyd and Richerson 1988; Cant et al. 2010; Fischer et al. 2014; Hardin 1982).

Heterogeneous resource environments could additionally favor cooperation because of increased relatedness in productive patches relative to unproductive patches. If dispersal rates from patches are relatively low, then most members of a patch will be residents rather than immigrants (Hamilton 1964; Griffin and West 2002; Lion and van Baalen 2008). Productive patches will, by definition, have a larger number of residents relative to immigrants (which come from both productive and unproductive patches), increasing relatedness and hence selection for cooperation within productive patches. This mechanism is unlikely to be particularly important in our experimental set up, because patches are completely recolonized at every transfer, hence on average the immigration rate is 50%.

The results may have more specific and applied relevance. *Pseudomonas aeruginosa* is an opportunistic human pathogen, particularly in nosocomial contexts, where it causes acute infection in immunocompromised and cystic fibrosis (CF) patients (Bodey et al. 1983). Cooperative traits such as siderophore production in *P. aeruginosa* are often linked with virulence (Harrison et al. 2006; Meyer et al. 1996; Takase et al. 2000). The CF lung environment is highly heterogeneous, both spatially and nutritionally, resulting in rapid diversification of *P. aeruginosa* within the CF lung (Jorth et al. 2015; Schick and Kassen 2018). This structured environment can result in between patch migration, where the most productive groups are successful in colonizing new parts of the lung. The role of this heterogeneity in maintaining cooperation (and hence virulence) will depend on the strength of hard versus soft selection in each case. However, the rate of between‐patch migration in the CF lung maybe be reduced due to mucous secreted from the host as well as biofilm‐secreting microbes. Increasing our understanding of how microbes function in ecological space and how this can in turn affect virulence will aid in reducing disease emergence, severity, and potential spread.

Here, we show that resource heterogeneity can allow a highly productive source population of cooperators to maintain cooperation at a higher level than a homogenous environment with the same average resource level. We have also shown theoretically that this effect—due to different populations having different productivities in a heterogeneous, metapopulation setting—disappears when between‐population differences in productivity are abolished by soft selection. More generally, our results emphasize the role of abiotic variation for the evolution and maintenance of cooperation.

Associate Editor: K. Lythgoe
